# Supplementary material for: Discovery of a Roman Quarry for Pozzolanic aggregates in the Euganean Hills Magmatic District, Northeast Italy: A stepwise archaeometric approach
Source: PLoS One. 2026 Apr 13;21(4):e0347202. doi: 10.1371/journal.pone.0347202 (PMC13075682; doi:10.1371/journal.pone.0347202)

**S1 Fig. XRPD patterns of the volcanic breccia clasts from mortar samples and quarry samples of Villa Draghi and Via Scagliara di M. Castellone.**

Mineral phases are labelled according to [61] (when mentioned): Sme = smectite; Bt = biotite; Amp = amphibole (horneblende type); Gp = gypsum; Sa = sanidine; Qz = quartz; Ano = anorthoclase; Pl = plagioclase; Cal = calcite; Cpx = clinopyroxene; Ilm = ilmenite; Mag = magnetite; Znc = zincite (internal standard).


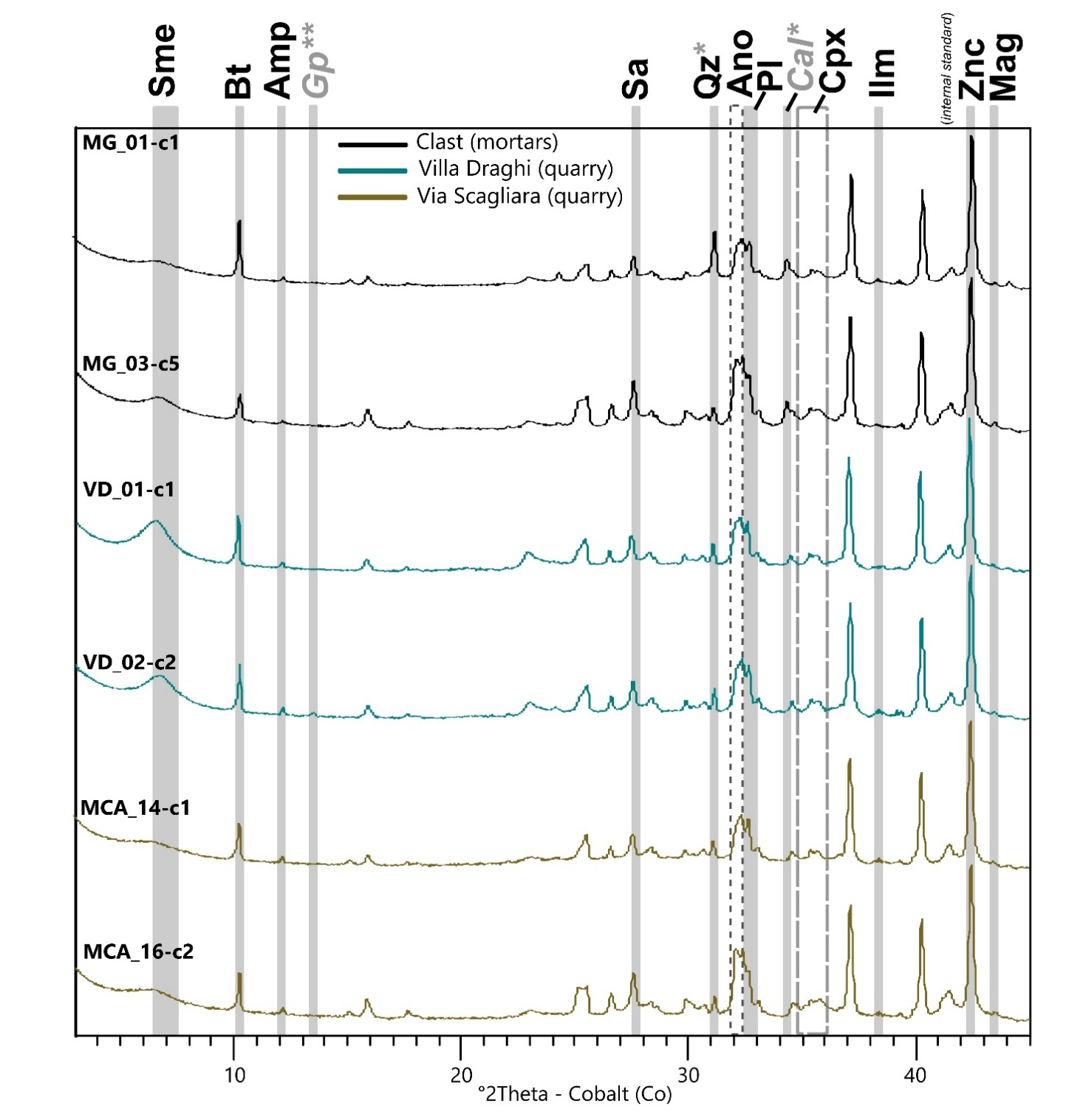

Supplement: S1 Fig — Mineral phases are labelled according to [61] (when mentioned): Sme = smectite; Bt = biotite; Amp = amphibole (horneblende type); Gp = gypsum; Sa = sanidine; Qz = quartz; Ano = anorthoclase; Pl = plagioclase; Cal = calcite; Cpx = clinopyroxene; Ilm = ilmenite; Mag = magnetite; Znc = zincite (internal standard). (DOCX) [file pone.0347202.s001.docx]
