# Supplementary material for: Discovery of a Roman Quarry for Pozzolanic aggregates in the Euganean Hills Magmatic District, Northeast Italy: A stepwise archaeometric approach
Source: PLoS One. 2026 Apr 13;21(4):e0347202. doi: 10.1371/journal.pone.0347202 (PMC13075682; doi:10.1371/journal.pone.0347202)
Supplement: S1 File — (DOCX) [file pone.0347202.s002.docx]

**S1 File. Instrumental equipment and standards.**

*Transmitted Polarized Light Optical Microscopy (TPL-OM).*

All mortar samples were analyzed by means of Transmitted Polarized Light – Optical Microscopy (*TPL-OM*) on 30 μm thin sections under a Leica DM790 P equipped with an integrated digital camera FLEXACAM I5 working with objective magnifications at 1.6x, 2.5x, 4x, 10x, 20x.

*Quantitative Phase Analysis - X-Ray Powder Diffraction (QPA-XRPD).*

QPA-XRPD analyses were performed on the bulk mortar samples and quarry samples, mechanically ground in an agate mortar. Moreover, QPA-XRPD analyses were also performed on the binder-concentrated material from the samples separated in water solution following the Cryo2Sonic 2.0 separation procedure [1], custom-modified by the addition of a chelating agent (sodium hexametaphosphate 0.5 %wt) to favour the suspension of the finer, non-carbonate phases such as clay minerals and hydrate products, prone to flocculation due to their surface charges. XRPD profiles were collected using a Bragg–Brentano θ-θ diffractometer (PANalytical X’Pert PRO, Cu Kα radiation, 40 kV and 40 mA) equipped with a real-time multiple strip (RTMS) detector (PIXcel by Panalytical). Data acquisition was performed by operating a continuous scan in the 3–85 [◦2θ] range, with a virtual step scan of 0.02 [◦2θ]. Diffraction patterns were interpreted with X’Pert HighScore Plus 3.0 software by PANalytical, qualitatively reconstructing mineral profiles of the compounds by comparison with PDF databases from the International Centre for Diffraction Data (ICDD). Quantitative phase analysis (QPA) was performed using the Rietveld method [2]. Refinements were carried out with TOPAS software (version 4.1) by Bruker AXS. The quantification of both crystalline and amorphous content was obtained through the addition of 20 %wt. of zincite to the powders as internal standard. The observed Bragg peaks in the powder patterns have been modelled through a pseudo-Voigt function, fitting the background with a 12 coefficients Chebyshev polynomial. For each mineral phase, lattice parameters, Lorentzian crystal sizes and scale factors have been refined. Although samples were prepared with the backloading technique to minimize preferred orientation of crystallites a priori, any residual preferred orientation effect was modelled during the refinement with the March Dollase algorithm. The starting structural models for the refinements were taken from the International Crystal Structure Database (ICSD).

*Scanning Electron Microscopy (SEM) coupled with Energy-Dispersive X-Ray Spectroscopy (EDS).*

SEM-EDS analyses were conducted to investigate the local chemical composition of both binder and aggregates, as well as the reaction zones within the samples. Analyses were performed using a FEI Quanta 200 scanning electron microscope equipped with an EDAX Element-C2B Energy Dispersive X-ray (EDX) detector. This instrument was employed for both microchemical and microstructural characterization of the mortar samples, prepared as 30 µm thin sections following the previously described separation procedure and embedded in resin. The mortars’ thin sections were carbon-coated prior to EDS analysis to ensure optimal conductivity and analytical accuracy.

Chemical compositions of crystals in the analyzed volcanic rocks, both from mortars and geological samples, were determined on polished 30 μm thin sections using a Tescan Solaris field-emission scanning electron microscope (FE-SEM) equipped with an Ultim Max 65 energy-dispersive X-ray spectroscopy (EDS) silicon drift detector (SDD) from Oxford Instruments. This EDS, thanks to the SDD technology and the big active sensor area (65 mm^2^), provides high resolution performances and accurate quantitative analyses due to a high signal-to-noise ratio and high-count rates up to 400.000 cps (count per second). In addition, the Ultim Max 65 has a high spectral resolution, in particular the instruments we used has a spectral resolution of 124 eV at MnKa up to 200.000 cps. The EDS values have been standardized using the Smithsonian microbeam standards and the ones provided by Cameca. Standards were both natural minerals and artificial oxides, we used: Natural Bridge diopside (Ca), San Carlos olivine (Mg), Amelia plagioclase (Na, Al, Si), iron oxide (Fe), pyrophanite (Mn and Ti), orthoclase (K), chromium oxide (Cr), nickel oxide (Ni), barite (S, Ba). For amphibole analyses we used the Kakanui hornblende as a standard for Al, Si and Mg. Analytical conditions for chemical analyses were 15 KeV-3 nA with a working distance of 5 mm. Each EDS point analyses have been acquired for 20 seconds, and the current was calibrated using a cobalt reference. ZAF correction built in the Oxford AzTec software has been also applied.

Standard analysis (values expressed in oxides)

|  | **Amelia Plagioclase** | |  |
| --- | --- | --- | --- |
|  | *Expected* | *Mean* | *St. dev.* |
| Na_2_O | 11,46 | 11,44 | 0,07 |
| Al_2_O_3_ | 19,76 | 19,75 | 0,03 |
| SiO_2_ | 68,14 | 68,34 | 0,08 |
| K_2_O | 0,23 | 0,22 | 0,03 |
| CaO | 0,38 | 0,07 | 0,02 |
| Tot | 99,97 | 99,82 |  |

|  | **Natural Bridge Diopside** | | |
| --- | --- | --- | --- |
|  | *Expected* | *Mean* | *St. dev.* |
| Na_2_O | 0,25 | 0,16 | 0,01 |
| MgO | 17,79 | 18,08 | 0,04 |
| Al_2_O_3_ | 0,11 | 0,21 | 0,02 |
| SiO_2_ | 55,81 | 56,01 | 0,14 |
| CaO | 25,28 | 25,33 | 0,04 |
| MnO | 0,04 | 0,03 | 0,04 |
| FeO | 0,25 | 0,28 | 0,03 |
| Tot | 99,53 | 100,10 |  |

|  | **San Carlos Olivine** | |  |
| --- | --- | --- | --- |
|  | *Expected* | *Mean* | *St. dev.* |
| MgO | 49,42 | 49,52 | 0,04 |
| SiO_2_ | 40,81 | 40,77 | 0,08 |
| MnO | 0,14 | 0,17 | 0,03 |
| FeO | 9,55 | 9,34 | 0,06 |
| NiO | 0,37 | 0,43 | 0,06 |
| Tot | 100,29 | 100,23 |  |

|  | **Hornblende Kakanui** | | |
| --- | --- | --- | --- |
|  | *Expected* | *Mean* | *St. dev.* |
| Na_2_O | 2,60 | 2,46 | 0,01 |
| MgO | 12,80 | 12,85 | 0,04 |
| Al_2_O_3_ | 14,90 | 14,99 | 0,05 |
| SiO_2_ | 40,37 | 40,74 | 0,05 |
| K_2_O | 2,05 | 2,03 | 0,01 |
| CaO | 10,30 | 9,85 | 0,06 |
| TiO_2_ | 4,72 | 4,88 | 0,07 |
| MnO | 0,09 | 0,11 | 0,04 |
| FeO | 7,95 | 10,90 | 0,06 |
| Fe_2_O_3_ | 3,30 |  |  |
| Tot | 99,08 | 98,81 |  |

|  | **Orthoclase** | |  |
| --- | --- | --- | --- |
|  | *Expected* | *Mean* | *St. dev.* |
| Na_2_O | 0,44 | 0,38 | 0,02 |
| Al_2_O_3_ | 17,17 | 17,30 | 0,02 |
| SiO_2_ | 65,36 | 64,96 | 0,04 |
| K_2_O | 15,70 | 15,75 | 0,05 |
| Fe_2_O_3_ | 1,24 | 1,08 | 0,06 |
| Tot | 99,91 | 99,47 |  |

|  | **MnTiO_3_** |  |  |
| --- | --- | --- | --- |
|  | *Expected* | *Mean* | *St. dev.* |
| TiO_2_ | 52,96 | 52,95 | 0,06 |
| MnO | 47,04 | 47,07 | 0,14 |
| Tot | 100,00 | 100,02 |  |

|  | **Fe_2_O_3_** |  |  |
| --- | --- | --- | --- |
|  | *Expected* | *Mean* | *St. dev.* |
| O | 30,06 | 29,84 | 0,15 |
| Fe | 69,94 | 70,16 | 0,15 |
| Tot | 100,00 | 100,00 |  |

|  | **NiO** |  |  |
| --- | --- | --- | --- |
|  | *Expected* | *Mean* | *St. dev.* |
| O | 21,42 | 21,40 | 0,03 |
| Ni | 78,57 | 78,55 | 0,12 |
| Tot | 99,99 | 99,95 |  |

|  | **BaSO_4_** |  |  |
| --- | --- | --- | --- |
|  | *Expected* | *Mean* | *St. dev.* |
| SO_3_ | 34,30 | 34,28 | 0,06 |
| BaO | 65,70 | 65,69 | 0,14 |
|  | 100,00 | 99,97 |  |

|  | **Cr_2_O_3_** |  |  |
| --- | --- | --- | --- |
|  | *Expected* | *Mean* | *St. dev.* |
| O | 31,58 | 31,59 | 0,02 |
| Cr | 68,42 | 68,45 | 0,04 |
|  | 68,42 | 100,04 |  |

Detection limits

| **Plagioclase (Plg)** | | | **Amphibole (Amp)** | | **Biotite (Bt)** | | | **Magnetite (Mag)** | |
| --- | --- | --- | --- | --- | --- | --- | --- | --- | --- |
| *Oxide* | *Oxide %*  *Sigma* | | *Oxide* | *Oxide*  *% Sigma* | *Oxide* | *Oxide*  *% Sigma* | | *Element* | *Wt% Sigma* |
| Na_2_O | 0,04 | | Na_2_O | 0,04 | Na_2_O | 0,03 | | O | 0,09 |
| Al_2_O_3_ | 0,08 | | MgO | 0,06 | MgO | 0,06 | | Na | 0,03 |
| SiO_2_ | 0,13 | | Al_2_O_3_ | 0,06 | Al_2_O_3_ | 0,07 | | Mg | 0,03 |
| K_2_O | 0,02 | | SiO_2_ | 0,11 | SiO_2_ | 0,1 | | Al | 0,03 |
| CaO | 0,05 | | K_2_O | 0,03 | K_2_O | 0,04 | | Si | 0,02 |
| FeO | 0,05 | | CaO | 0,06 | CaO | 0,03 | | P | 0,02 |
|  |  |  | TiO_2_ | 0,06 | TiO_2_ | 0,07 | | K | 0,02 |
|  |  |  | MnO | 0,05 | MnO | 0,05 | | Ca | 0,02 |
|  |  |  | FeO | 0,11 | FeO | 0,11 | | Ti | 0,04 |
|  |  |  | BaO | 0,1 | BaO | 0,11 | | V | 0,03 |
|  |  |  |  |  |  |  |  | Cr | 0,03 |
|  |  |  |  |  |  |  |  | Mn | 0,05 |
|  |  |  |  |  |  |  |  | Fe | 0,17 |
|  |  |  |  |  |  |  |  | Co | 0,08 |
|  |  |  |  |  |  |  |  | Ni | 0,06 |

*X-Ray Fluorescence*

The XRF analyses were performed on volcanic rock samples with a WDS Panalytical Zetium sequential spectrometer, operating under vacuum conditions, equipped with a 2.4-kW Rh tube. The samples were calcined to determine their loss on ignition (L.O.I.) by placing them in a muffle furnace at 860 °C for about 20 min, and then at 980 °C for about 2 h. The samples for the actual XRF analyses were then prepared in beads using lithium tetraborate (Li_2_B_4_O_7_) flux with a dilution of 1:10, and melting was carried out using a Claisse Eagon 2 bead mill (maximum temperature achieved of about 1150 °C). The calculated major elements are Si, Ti, Al, Fe, Mn, Mg, Ca, Na, K, and P (expressed as percentages of the relative oxides). The L.O.I. was calculated separately. The calculated trace elements (expressed in ppm) are Sc, V, Cr, Co, Ni, Cu, Zn, Ga, Rb, Sr, Y, Zr, Nb, Ba, La, Ce, Nd, Pb, Th, and U. Instrumental precision (defined by repeated analyses on the same sample) is within 0.6% relative for major elements and within 3.0% relative for trace elements. Detection limits for Al, Mg, and Na are within 0.01%, within 0.2% for Si, and within 0.005% for Ti, Fe, Mn, Ca, K and P; for trace elements, they are (in ppm): Sc = 3, V = 5, Cr = 6, Co = 3, Ni = 3, Cu = 3, Zn = 3, Ga = 3, Rb = 3, Sr = 3, Y = 3, Zr = 3, Nb = 3, Ba = 10, La = 10, Ce = 10, Nd = 10, Pb = 5, Th = 3, U = 3.

For the quarry reference samples, bulk analyses were conducted on coarse-grained material, with 20.0 g of powder prepared per sample sifted at a grain size ≤ 180 µm. Volcanic clasts embedded in the mortars were mechanically separated from the matrix, and only the coarser fragments weighing more than 2.0 g were prepared according to the same procedure and analyzed. Prior to analysis, mortars’ volcanic clasts were cleaned mechanically to remove as much as possible the reaction rim at the clast-binder interface. They were subsequently subjected to a 3% HCl bath for 24 hours to further eliminate carbonates from the lime. It is important to note that this acid treatment can induce leaching of certain phases, potentially altering the concentrations of mobile elements, particularly Y [3]. Consequently, Y was excluded from comparative analyses with quarry reference samples. For matching determination, selected chemical elements from XRF data were plotted in bivariate scatterplots for visual evaluation of distribution of reference geological samples in comparison with archeological ones. Selected chemical elements, useful for provenance determination, were also treated via statistical analyses (e.g. Linear Discriminant Analysis, LDA) using the integrated tools of Statgraphics Centurion Pro 19, as reported in [4].

*µ-Raman*

µ-Raman analyses were performed on magnetite crystals to verify their exact mineralogy and to identify possible secondary phases. The analyses were carried out using a WITec Alpha 300R Raman system (WITec GmbH) equipped with a Zeiss microscope and a 532 nm laser. Spectra of magnetites were acquired with the Control Five programme (®WITec) in the range 100–1500 cm^-1^, with grating 300 groove/mm, magnification 100X, power 5.0 mW, using an integration time of 1 second and 60 accumulations.

**References**

1. Addis A., Secco M, Marzaioli F., Artioli G., Chavarria Arnau A., Passariello I., Terrasi F., Brogiolo G.P. 2019 et al. Selecting the most reliable ¹⁴C dating material inside mortars: The origin of the Padua cathedral. Radiocarbon 2019, 61: 375–393.
2. Rietveld HM. Line profiles of neutron powder-diffraction peaks for structure refinement. Acta Crystallogr. A 1967; 22: 151-152.
3. Marra F, Anzidei M, Benini A, D’Ambrosio E, Gaeta M, Ventura G, Cavallo A. Petro-chemical features and source areas of volcanic aggregates used in ancient Roman maritime concretes. J. Volcanol. Geotherm. Res. 2016; 328: 59-69. doi: 10.1016/j.jvolgeores.2016.10.005.
4. Dilaria S., Ricci G., Secco M., Beltrame C., Costa E. Giovanardi T., Bonetto J., Artioli G. Vitruvian binders in Venice: First evidence of Phlegraean pozzolans in an underwater Roman construction in the Venice Lagoon. *PLoS ONE* 2024, 19: e0313917.
