# Supplementary material for: Discovery of a Roman Quarry for Pozzolanic aggregates in the Euganean Hills Magmatic District, Northeast Italy: A stepwise archaeometric approach
Source: PLoS One. 2026 Apr 13;21(4):e0347202. doi: 10.1371/journal.pone.0347202 (PMC13075682; doi:10.1371/journal.pone.0347202)

**S2 Fig. Selected discriminant scatterplots for Villa Draghi and Via Scagliara di M. Castellone quarries.** The archaeological clasts exhibit intermediate distribution among the quarry intervals.


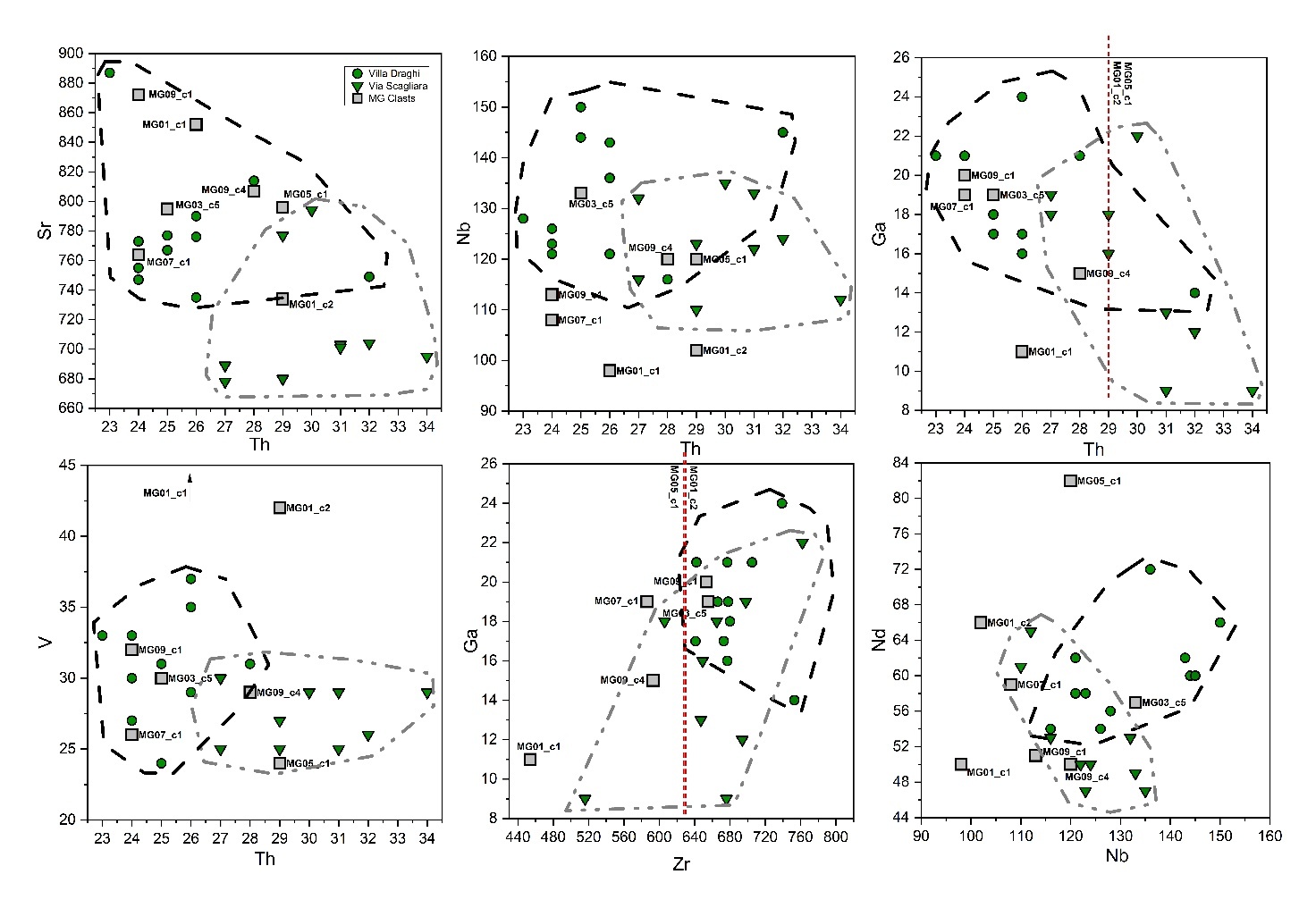

Supplement: S2 Fig — The archaeological clasts exhibit intermediate distribution among the quarry intervals. (DOCX) [file pone.0347202.s004.docx]
