# Supplementary material for: Discovery of a Roman Quarry for Pozzolanic aggregates in the Euganean Hills Magmatic District, Northeast Italy: A stepwise archaeometric approach
Source: PLoS One. 2026 Apr 13;21(4):e0347202. doi: 10.1371/journal.pone.0347202 (PMC13075682; doi:10.1371/journal.pone.0347202)
Supplement: S3 Fig — Sample VD_02 from Villa Draghi Quarry (Spectra 1–4); sample MCA_16 (Spectrum 5). (DOCX) [file pone.0347202.s006.docx]

**S3 Fig. µ-Raman spectra of magnetite crystals, exhibiting diagnostic peaks attributable to both magnetite and maghemite (maghemitized magnetite).** Sample VD_02 from Villa Draghi quarry (Spectra 1-4); sample MCA_16 (Spectrum 5) from Via Scagliara of M. Castellone quarry.
