# Supplementary material for: Discovery of a Roman Quarry for Pozzolanic aggregates in the Euganean Hills Magmatic District, Northeast Italy: A stepwise archaeometric approach
Source: PLoS One. 2026 Apr 13;21(4):e0347202. doi: 10.1371/journal.pone.0347202 (PMC13075682; doi:10.1371/journal.pone.0347202)

**S5 Fig. K_2_O vs Zr, K_2_O vs Na_2_O and K_2_O vs Al_2_O_3_ scatterplots used for provenance discrimination of the archaeological volcanic rock clasts from the foundational mortars of the Great Baths of Aquileia.** The reference clusters with homogeneous samples from the same quarry sites are marked by dashed or dotted and dashed lines. Geological marker samples are plotted from the UNIPD reference database.


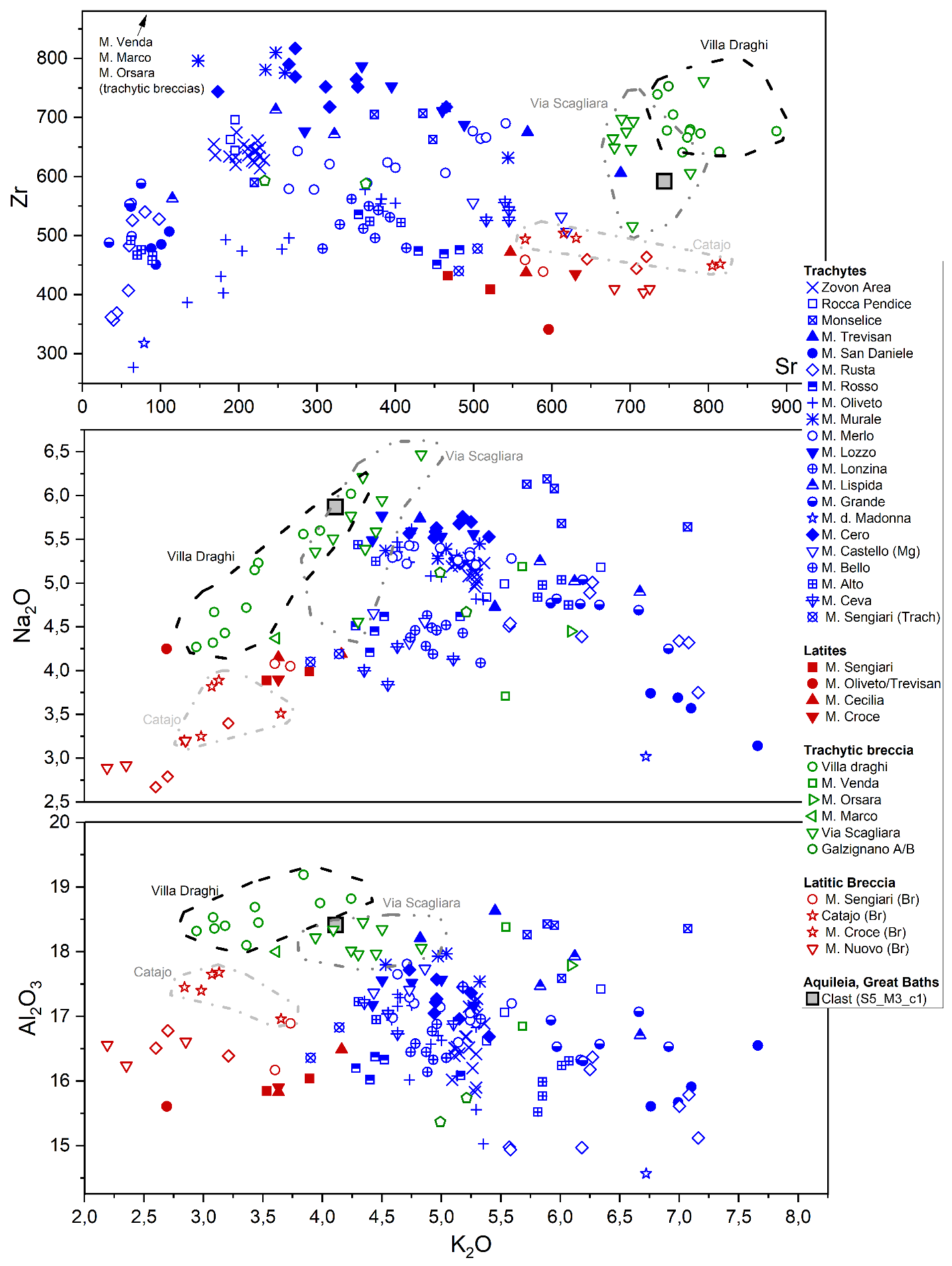

Supplement: S5 Fig — The reference clusters with homogeneous samples from the same quarry sites are marked by dashed or dotted and dashed lines. Geological marker samples are plotted from the UNIPD reference database. (DOCX) [file pone.0347202.s008.docx]
