# Supplementary material for: Discovery of a Roman Quarry for Pozzolanic aggregates in the Euganean Hills Magmatic District, Northeast Italy: A stepwise archaeometric approach
Source: PLoS One. 2026 Apr 13;21(4):e0347202. doi: 10.1371/journal.pone.0347202 (PMC13075682; doi:10.1371/journal.pone.0347202)
Supplement: S6 Fig — Sample GTR_S5_M3-c1, described in [80]. Legend: Ap = apatite. (DOCX) [file pone.0347202.s009.docx]

**S6 Fig. SEM-EDS analysis of magnetite crystals within the volcanic breccias included in the foundational mortars of the Great Baths of Aquileia.** Sample GTR_S5_M3-c1, described in [80]. Legend: Ap = apatite.

| 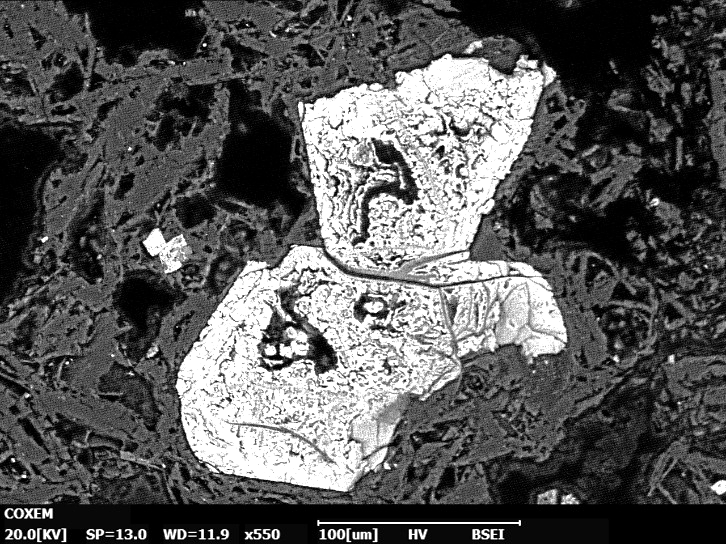 | 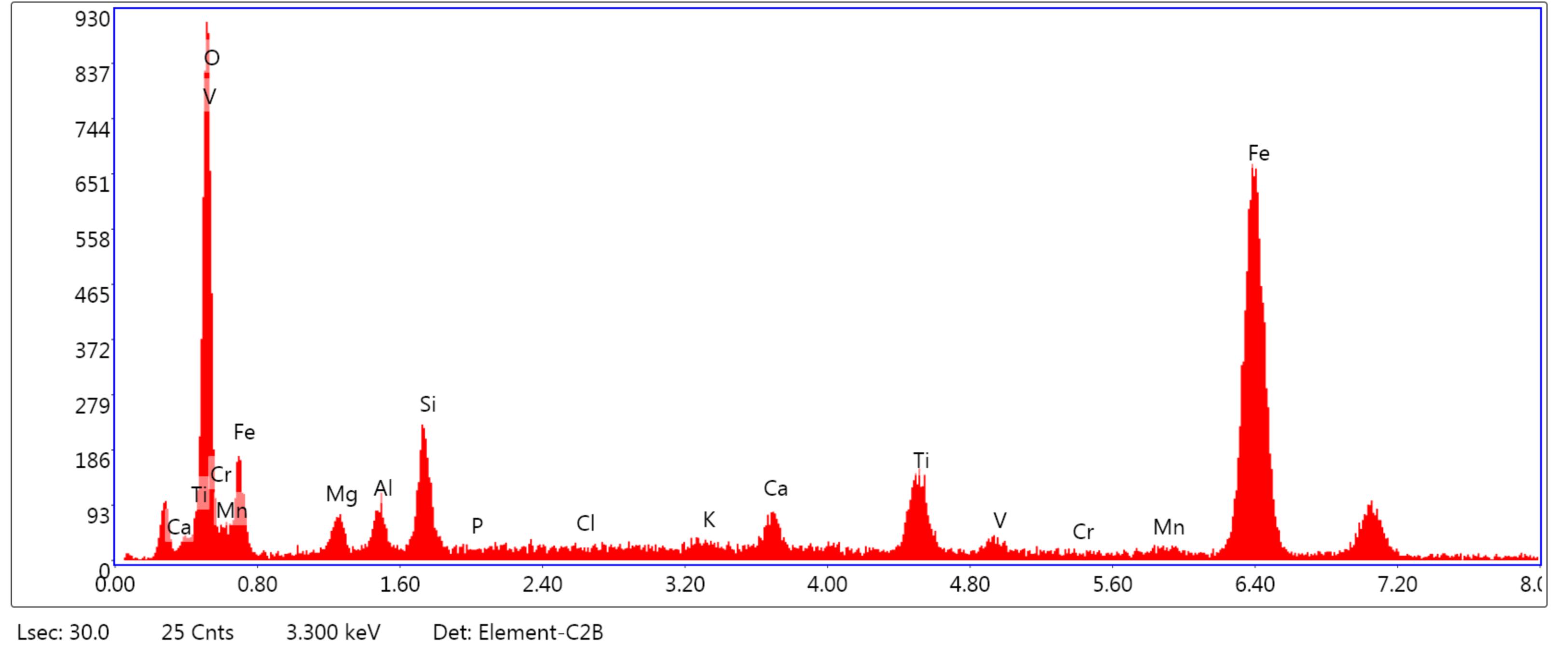 |
| --- | --- |
| 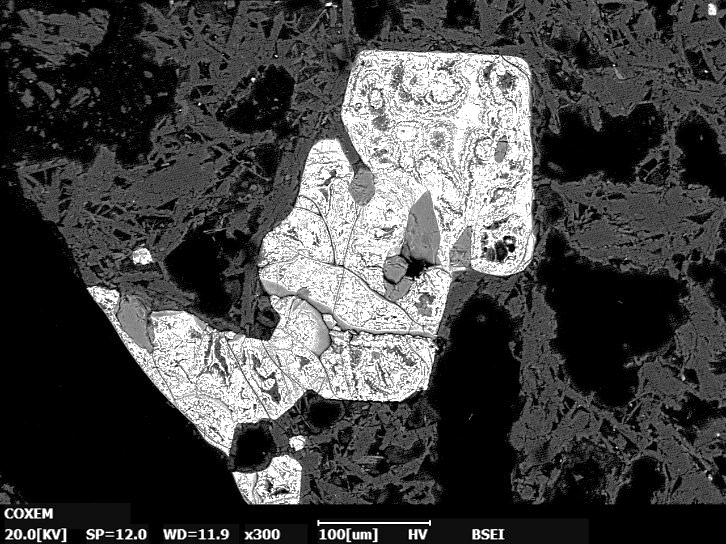  ***Ap*** | 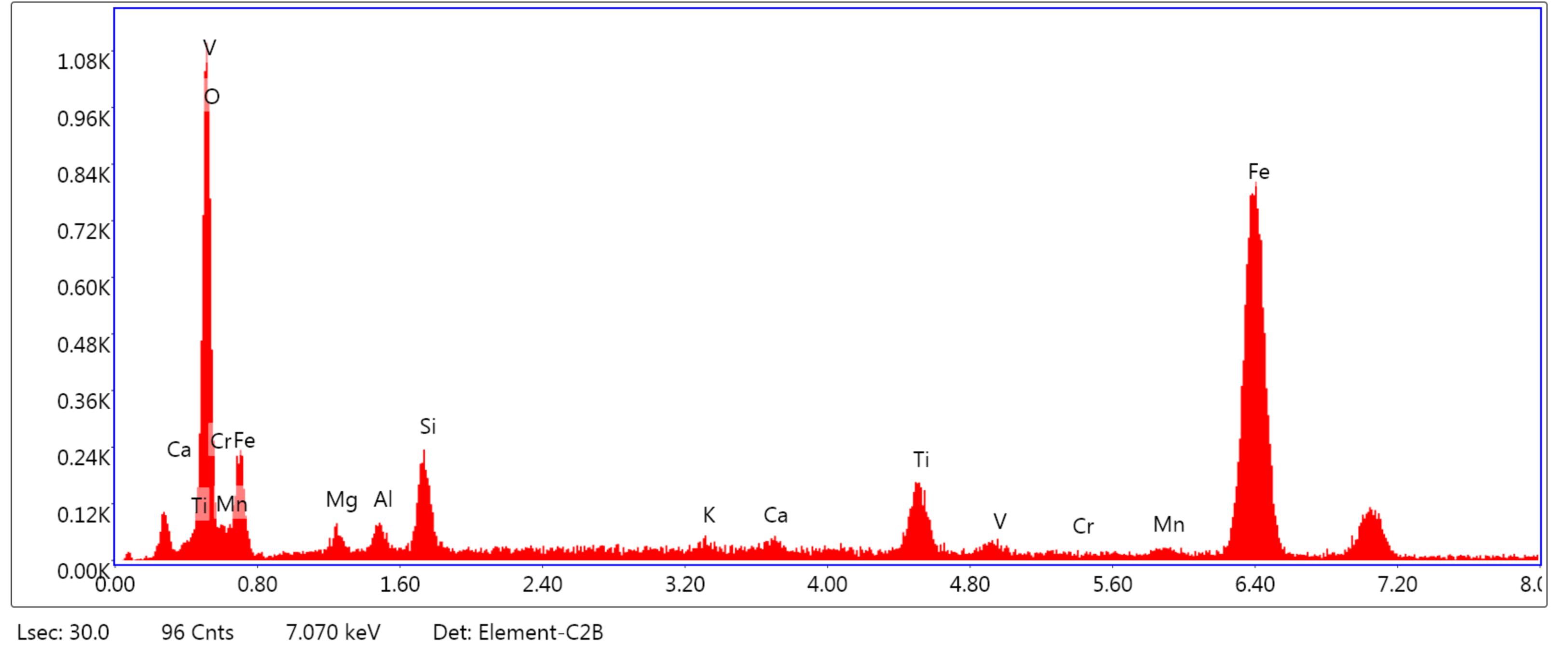 |
| 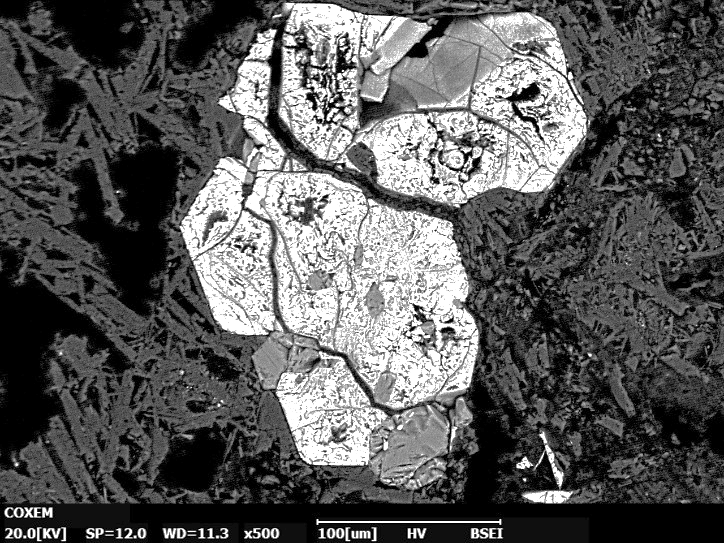  ***Ap*** | 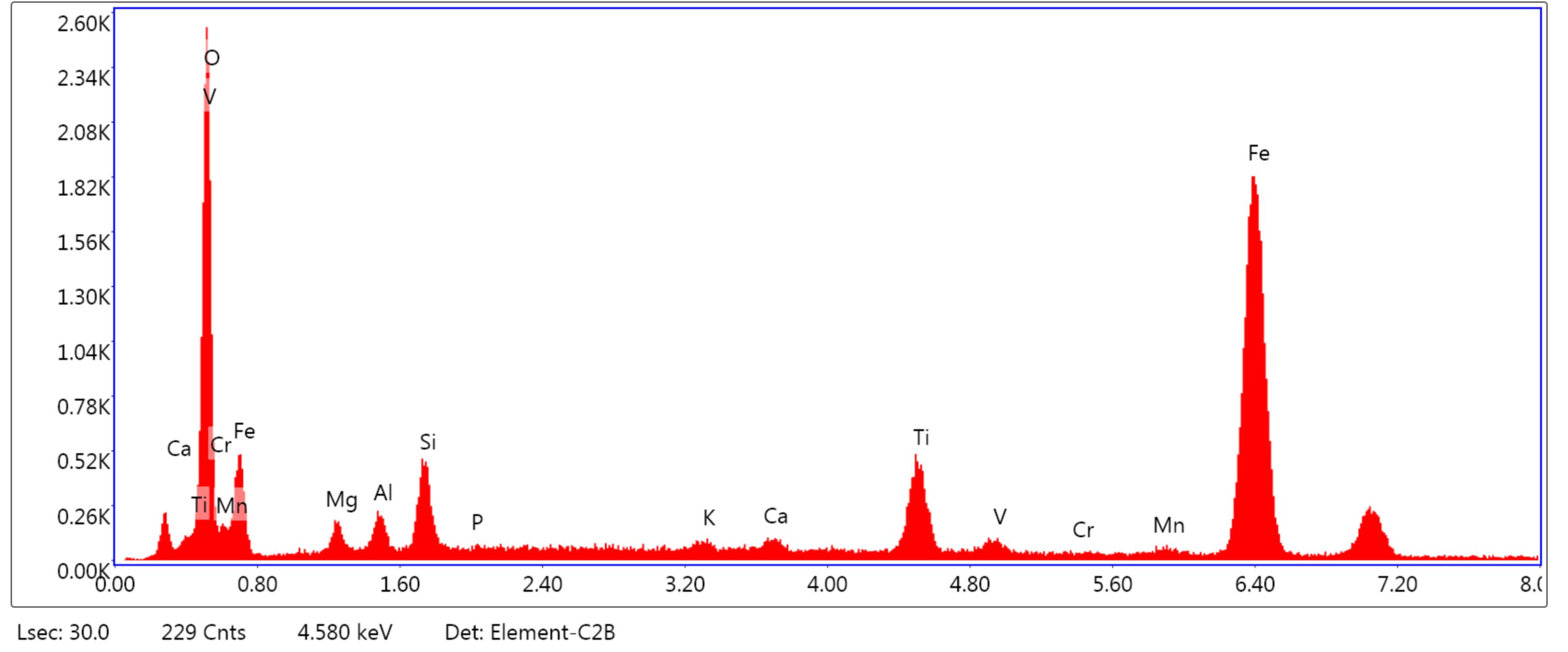 |
| 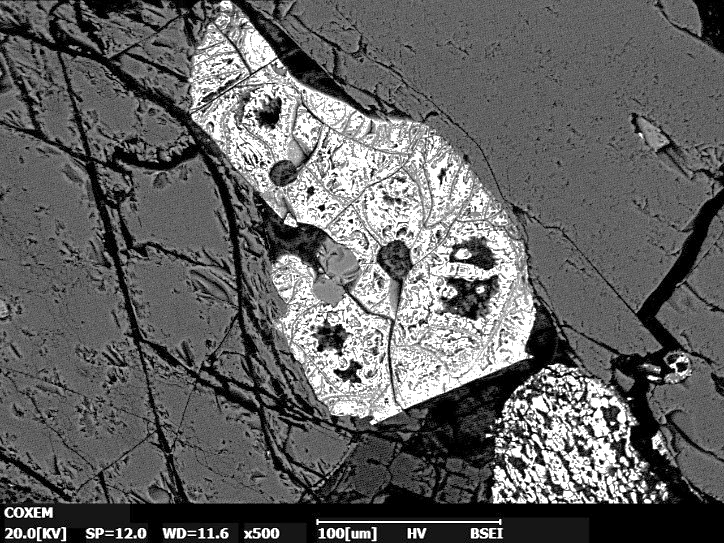  *Ap* | 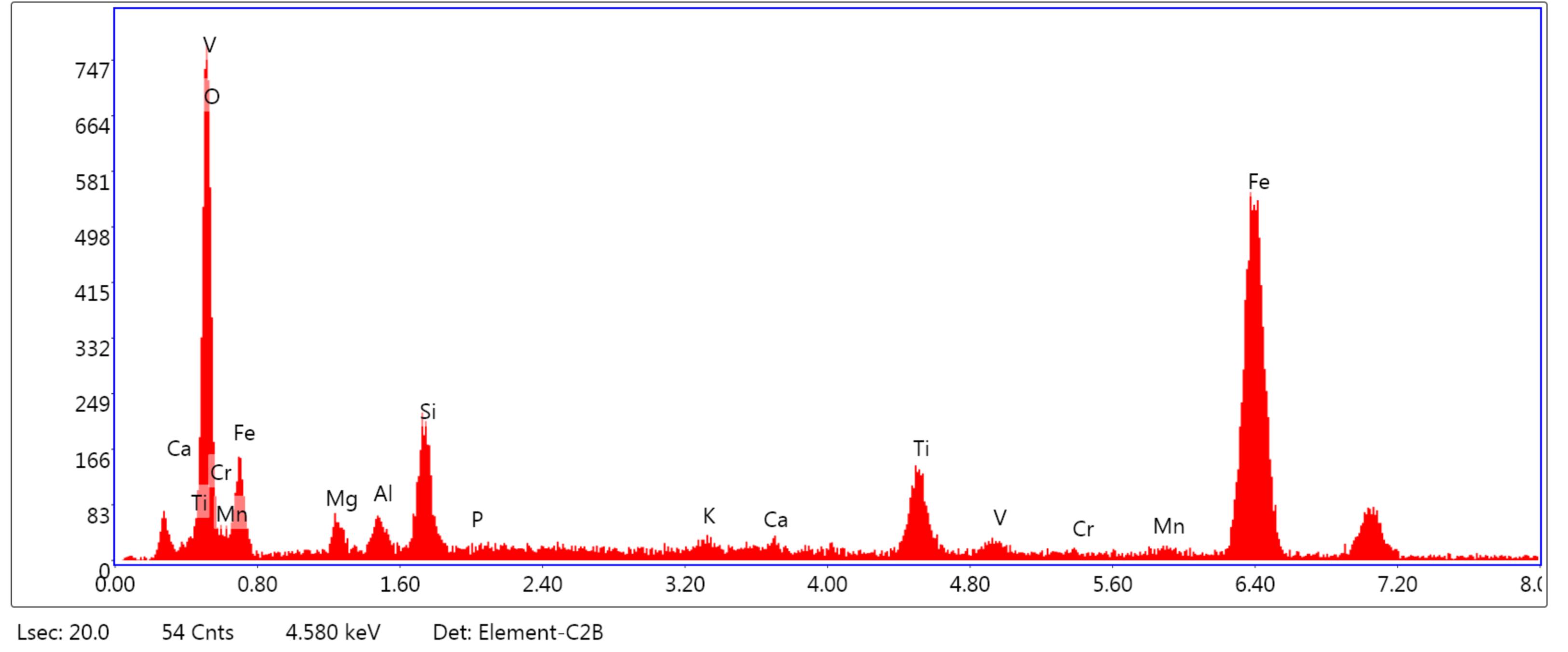 |
