# Supplementary material for: Discovery of a Roman Quarry for Pozzolanic aggregates in the Euganean Hills Magmatic District, Northeast Italy: A stepwise archaeometric approach
Source: PLoS One. 2026 Apr 13;21(4):e0347202. doi: 10.1371/journal.pone.0347202 (PMC13075682; doi:10.1371/journal.pone.0347202)
Supplement: Attachment — Submitted filename: Response to Reviewers_3.pdf [file pone.0347202.s010.pdf]

DIPARTIMENTO DEI BENI CULTURALI:
ARCHEOLOGIA, STORIA DELL'ARTE,
DEL CINEMA E DELLA MUSICA

Piazza Capitaniato 7
35139 Padova

Padova, January 2026

To the Editors of PLoS ONE Journal,

please find attached our manuscript titled "Discovery of a Roman Quarry for Pozzolanic Aggregates in the Euganean Hills Volcanic District, Northeast Italy: an archaeometric approach", by myself and co-authors, for publication in PLoS ONE journal.

The observations have been thoroughly revised with a rebuttal for the point addressed by Gema Queenie Salem and Paula Katrina A. Maderazo

- Thank you for our response. However, it appears there is still a small map image included in Figure 1. Please clarify the source of this map image and its copyright status.

Figure 1 has been completely revised and clarified. The entire figure was produced by the authors of this paper. All polylines, drawings, inset maps, sample locations, sample labels, colors, and the basemap were created exclusively using data generated by the authors.

Sincerely,

Michele Secco, Ph.D. (corresponding author)

Associate professor – GEOS-01 (Mineralogy, Petrology, Geochemistry, Volcanology, Georesources and applications)

University of Padova, Department of Cultural Heritage

Piazzetta Folena, 1 - 35137 Padova (Italy)

michele.secco@unipd.it
