# Supplementary material for: Discovery of a Roman Quarry for Pozzolanic aggregates in the Euganean Hills Magmatic District, Northeast Italy: A stepwise archaeometric approach
Source: PLoS One. 2026 Apr 13;21(4):e0347202. doi: 10.1371/journal.pone.0347202 (PMC13075682; doi:10.1371/journal.pone.0347202)

dBC

Università degli Studi di Padova

DIPARTIMENTO DEI BENI CULTURALI:
ARCHEOLOGIA, STORIA DELL'ARTE,
DEL CINEMA E DELLA MUSICA

Piazza Capitaniato 7
35139 Padova

Padova, March 2026

To the Editors of PLoS ONE Journal,

please find attached the reviewed version of our manuscript with the slightly revised title "Discovery of a Roman Quarry for Pozzolanic Aggregates in the Euganean Hills Magmatic District, Northeast Italy: a stepwise archaeometric approach" by myself

and co-authors, for publication in PLoS ONE Journal.

The answers to the comments of both the two reviewers and editor are reported hereafter (in italics).

Academic Editor

- Concerning XRF sample preparation, mortar clasts were treated with 3% HCl for 24 hours prior to analysis. While you note the possible effect on Y and exclude it from comparative analyses, please clarify whether the potential impact of acid treatment on other mobile elements was evaluated, and briefly state how you ensured that the trace element signature used for provenance comparison was not materially biased by the leaching procedure. Please also correct the typographical error "form XRF data" to "from XRF data".

  We thank the Reviewer for this important observation regarding the 3% HCl treatment. Our approach was based on a conservative selection of tracers to guarantee the reliability of the provenance data. Being aware that the exact extent of leaching cannot be quantified for every element, we proactively excluded Yttrium (Y) from all discriminant models, as it appeared most susceptible to mobilization, as observed in similar case studies tailored to the analyses of volcanic aggregates included in mortars (e.g. D'Ambrosio et al. 2015; Marra et al. 2016). In fact, the provenance analysis relied on elements characterized by lower geochemical mobility (e.g., Zr, Nb, Th), which are hosted within stable silicate lattices or resistant accessory minerals. These phases are significantly less affected by mild acid leaching than elements associated with the carbonatic binder. Moreover, the suitability of the selected tracers is empirically validated by the high statistical coherence of the archaeological dataset. In case the leaching procedure would have been materially biased the results, we would have observed high dispersion ("geochemical noise") and a lack of correlation with the sources. Instead, the archaeological clasts maintained sharp clustering and strong affinity with the geological references.

  Typographical error "form XRF data" to "from XRF data": corrected.

- You report the use of Linear Discriminant Analysis for provenance discrimination. Please indicate whether any form of model validation (e.g. leave-one-out or cross-validation) was performed and briefly report the outcome. This will help readers assess the robustness of the discriminant model and interpret the reported classification performance appropriately.

  We thank the editor for this pertinent suggestion. To ensure the highest degree of statistical transparency and to assess the robustness of our provenance assignments, we have further validated the Linear Discriminant Analysis (LDA) using Leave-One-Out Cross-Validation (LOOCV). This procedure yielded a 100% correct classification rate for the reference training set

(M. Castellone vs. Villa Draghi) in both the "full variable" and "stepwise forward" configurations. This confirms that the geochemical separation between the two source quarries is exceptionally sharp and is not an artifact of overfitting. However, to provide a deeper assessment of the archaeological samples (MG group), we intentionally compared the results of the Full Model (10 variables) with a Stepwise Forward Model. While the reference sources remained stable (100% accuracy), this comparison highlighted the sensitivity of specific archaeological clasts (e.g., MG09_c4 and MG05_c1) to different statistical configurations. Therefore, we have clarified in the manuscript that these divergent outcomes for certain clasts are not merely statistical artifacts but stem from inherent material-specific ambiguities. Factors such as the limited size and heterogeneity of the clasts, the analytical resolution of bulk XRF, and post-depositional pozzolanic alterations can deviate geochemical signatures from their primary values.

We have profoundly revised this part of the manuscript comparing different LDA algorithms. Crucially, the observed variability in provenance attribution between the two models does not stem from statistical instability, but rather from the intrinsic nature and 'quality' of the processed archaeological data. Regardless of the cross-validation outcomes, the resolution of provenance assignments is fundamentally constrained by the geochemical representativeness of the bulk clast profile. At this analytical level, the primary signature of an archaeological sample can be significantly influenced by factors such as its limited size within the mortar matrix and its susceptibility to post-depositional alterations. In essence, the fluctuations in posterior probabilities reflect the 'noise' inherent to the archaeological material itself, where pozzolanic reactions and heterogeneity can deviate the clast from its pristine geological parent, rather than a flaw in the LDA modeling. Consequently, the statistical ambiguity is a transparent reflection of the material's complexity.

- There appears to be an inconsistency in the reported Raman laser power (3.0 mW versus 5 mW). Please clarify and ensure consistency throughout the manuscript and Supporting Information.

  Edited and corrected in the supporting file.

- Where magnetite/maghemite discrimination is discussed, please ensure that the evidential basis is presented consistently across methods (Raman and, where relevant, XRPD) and that the role of each technique is clearly stated.

  We thank the reviewer for this helpful comment. In the revised manuscript we have clarified the evidential basis used to discuss the discrimination between magnetite and maghemite and the specific role of each analytical technique. XRPD analyses identify magnetite as the dominant Fe-oxide phase in the samples. No maghemite reflections were detected in the diffraction patterns, which is likely due to its very low abundance and/or its occurrence as a minor alteration product below the detection limit of XRPD. Micro-Raman analyses, however, locally revealed spectral features consistent with maghemite, which we interpret as the result of partial oxidation of magnetite. To avoid ambiguity, the revised text now explicitly states that the term “magnetite” is used throughout the manuscript to refer to the main Fe-oxide phase identified by XRPD, while the occurrence of maghemite is discussed only in relation to the Raman data, where it is detected as a secondary alteration product.

- Finally, before resubmission, please carefully proofread the entire manuscript and Supporting Information to remove all editing artefacts and formatting remnants. The current version contains multiple traceable track-change/production artefacts (e.g. duplicated words, merged tokens, repeated “FigureFig” constructions, and residual formatting strings). These issues must be fully resolved in the next version, as they affect readability and presentation quality.

  We performed a complete polishing of the English in the text and we revised minor internal inconsistencies in the formatting and referencing of the paper

Reviewer 1

- I echo the academic editor’s comment, which advised authors to revise the keywords, so they complement rather than duplicate the title. The authors responded that they modified the keywords following the comment, but it seems to me that the keywords remain unchanged: Euganean Hills; Pozzolans; Roman mortars and binders; Provenance analysis; Roman Quarring and Trade.

  Edited and modified into: pozzolanic mortars, volcanic aggregates, geochemistry, Roman construction technology, stone provenance, magnetite

- The caption of the supplementary S2 Table "Results of SEM-EDS analyses on magnetites of breccia clasts from quarry sites of Villa Draghi and Via Scagliara and archaeological samples from Via Scavi in Montegrotto" should include all investigated minerals (Amphibole, Biotite, Plagioclases, Magnetites) names, as it only mentions magnetite.

  Edited and corrected

Reviewer 2

- Abstract: I already had a concern: aggregates sometimes define nor reactive ingredients, so calling them pozzolanic could be misleading.

  Edited and corrected

- Line 20: Punctual geochemical data by fluorescence?

  Corrected

- Line 21 (microRaman) instead of (mRaman) or use symbol

  Corrected along the entire text as μ-Raman

- Line 48: Unconsolidated volcanic tuffs: if using tuffs the term unconsolidated is wrong, it is better to use "non-lithified cineritic deposits"

  Corrected

- Line 48: NYT deposits are linked to CF, not to Somma-Vesuvius, so Authors should be consistent with volcanological features

  Corrected

- Line 55: Baia quarries are in the Gulf of Pozzuoli not in the Gulf of Naples

  Corrected along the text

- Line 59: Authors should be consistent: the term pozzolana indicates the unconsolidated part of the NYT, pozzolan(s) is more general term and indicates all volcanic deposits with pozzolanic activities.

  Corrected into volcanic pozzolans

- Line 73: … century CE, previously reported as c. BCE

  This is correct: the first evidence of local use points to the 2nd c BCE while the large diffusion occurs only during the Imperial period

- Line 90: once again pulvis puteolanus is from the Gulf of Pozzuoli

  Corrected along the text

- Table 1: Pozzolan type column reports seldom chemical compositions of the materials and could be sometimes misleading; please correct the term "revetments"

  Revetement corrected into coating; chemical definition of pozzolan types has been removed.

- Line 193: please clarify if lump refers to lime lumps

  Corrected

- Line 202: please remove the word stone

  Edited using the definition "lithic materials"

- Line 210: feldspar or plagioclase?

  We prefer to maintain the definition of feldspars as they comprise both plagioclases and scattered k-feldspars

- Line 214: please use carbonatic instead of carbonate

  Corrected

- Line 226: please add references for standard mortars

Removed the mention to standard mortars

- Line 227 vugs not vughs

  Corrected

- Line 249: no need to specify the valence of Mg (not previously reported for other cations)

  Corrected

- Line 267: please define Afm phases, not reported previously

  The sentence has been edited with a more complete definition of AFm phases

- Line 277-279: please do not use capital letter for minerals

  Done

- Table 3: The second decimal place (and the first too, IMHO) is completely useless.

  Corrected using only one decimal.

- Are errors available? Amorphous content was estimated by difference? Rietveld analyses of materials with such amount of amorphous is tricky, any further clarification (e.g. PONKS approach, peak phases addition) is available?

  The amorphous fraction was calculated using the internal standard approach, following the widely tested procedure described in S1 File.

- Line 361-374: It seems to me to be a repetition of what is stated in lines 200 to 211.

  This is the description of the geological samples, while before we were describing the clasts in the archaeological mortars. For the sake of clarity we preferred to provide both the descriptions highlighting the (minimal) differences between archaeological clasts and geological samples.

- 386-388: Authors should comment on presences, not absences.

  We added this sentence to highlight the main differences between the clasts and the Euganean volcanic lavas. We think that this sentence is useful to provide mineralogical elements of discrimination of the trachitic breccias in respect to the common lavas.

- Line 391: Augite is reported, please clarify its role and above all, its precise identification.

  We removed the mentioning of the augitic type.

- Line 393: Any other info on smectite type?

  We refined this phase using a smectite profile from BGMN. We suspect it is a montmorillonite type, but we did not clearly state this.

- Table 4: Gypsum is reported, not discussed in the text; please clarify how smectite was quantified.

  Gypsum is interpreted as a secondary phase formed through the sulphation of the exposed bedrock. Smectite was quantified using BGMN, applying refinements specifically tailored to improve the modelling of low-angle clay mineral reflections.

- Line 459: please define fresh minerals

  Removed fresh in the description of the minerals

- Line 485: see comment of line 21

  done

- Line 485-491: magnetite/maghemite could be clearly investigated by XRD (FWHM of the main peaks); any evidence through this technique?

  In the present study, magnetite was identified as the main Fe-oxide phase by Raman, although its reflections are weak and indicate a low abundance in the bulk samples. No clear evidence of maghemite was detected in the XRPD patterns, and therefore the discrimination between magnetite and maghemite based on peak position or FWHM was not possible. This is likely due to the very low concentration of Fe-oxide phases in the samples, which are close to the detection limit of XRPD. Evidence of maghemite was instead obtained only through point micro-Raman analyses, which locally revealed spectral features consistent with maghemite. These occurrences are interpreted as minor oxidation products derived from the

alteration of pristine magnetite. We have clarified this point in the revised manuscript, explicitly stating that maghemite was detected only locally by micro-Raman spectroscopy and was not identified by XRPD due to its very low abundance.

- Line 560: Please maintain consistency in the text with the term “Euganean pozzolans,” which is sometimes enclosed in quotation marks and sometimes not.

  Done, always adopted the quotation marks

- Supporting information: please do not use capital letters for minerals

  Done

- Line 888: see comment of line 21

  Done

Sincerely,

Michele Secco, Ph.D. (corresponding author)

Associate professor – GEOS-01 (Mineralogy, Petrology, Geochemistry, Volcanology, Georesources and applications)

University of Padova, Department of Cultural Heritage

Piazzetta Folena, 1 - 35137 Padova (Italy)

michele.secco@unipd.it

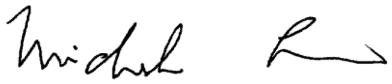

Supplement: Attachment — Submitted filename: Rebuttal letter_PLOS1.pdf [file pone.0347202.s011.pdf]
